# PHACE syndrome: a systematic literature review and illustrative case report of a patient with severe cerebrovascular and neurodevelopmental sequelae

**DOI:** 10.1007/s10072-026-09372-y

**Published:** 2026-09-04

**Authors:** Martina Gnazzo, Laura Caiazza, Caterina Sirocchi, Laura Beccani, Silvia Faccioli, Daniela Pandarese, Lorena Menta, Sara Gambretti, Benedetta Piccolo, Emanuela Turco, Elisa Incerti, Maria Carmela Pera

**Affiliations:** 1 Department of Mental Health, Physical and Preventive Medicine, Clinic of Child and Adolescent Neuropsychiatry, University of Campania “Luigi Vanvitelli”, Naples, Italy; 2 IRCCS Istituto Delle Scienze Neurologiche Di Bologna (ISNB), Bologna, Italy; 3 Department of Biomedical, Metabolic, and Neural Sciences, University of Modena and Reggio Emilia, Modena, Italy; 4 Department of Medicine and Surgery, University of Parma, Parma, Italy; 5 Unit for the Rehabilitation of Severe Developmental Disabilities, Azienda USL IRCCS of Reggio Emilia, Reggio nell’Emilia, Italy; 6 Unit of Rehabilitation Medicine, Azienda USL Di Parma, Parma, Italy; 7 Child Neuropsychiatric Unit, Maternal and Child Health Department, Parma University Hospital, 43126 Parma, Italy; 8 Child Neuropsychiatry Unit, Department of Medicine and Surgery, University of Parma, Parma, Italy

**Keywords:** PHACE syndrome, Infantile hemangioma, Cerebrovascular arteriopathy, Dural arteriovenous fistula, Hydrocephalus, Neurodevelopmental outcome, Systematic review

## Abstract

**Background:**

PHACE syndrome is a rare neurocutaneous disorder defined by the association of large segmental infantile hemangiomas of the head and neck with malformations of the posterior fossa, cerebral and cervical arteries, heart, eyes, and ventral midline structures. Although facial hemangiomas are often the presenting feature, the cerebrovascular, neurodevelopmental, and airway manifestations are responsible for the greatest long-term morbidity.

**Methods:**

A systematic literature review was conducted following the Preferred Reporting Items for Systematic Reviews and Meta-Analyses (PRISMA) guidelines, searching PubMed, Web of Science, EMBASE, and PsycINFO. After removal of duplicates and screening of 308 records, five studies meeting the inclusion criteria were retained for qualitative synthesis. We additionally present the case of a now 12-year-old girl with PHACE syndrome characterized by a left V1-distribution facial hemangioma, ocular abnormalities, multiple cerebrovascular venous and arterial malformations, neonatal intraventricular hemorrhage with hydrocephalus, and a subsequently diagnosed dural arteriovenous fistula requiring repeated embolization.

**Results:**

The five included studies collectively describe epidemiology and early supportive care needs, long-term health outcomes and quality of life into adulthood, airway hemangioma prevalence and management, and the clinical spectrum of infantile hemangiomas with minimal or arrested growth (IH-MAG) as a cutaneous marker of PHACE syndrome. Across studies, cerebrovascular arteriopathy (72–91%) and facial hemangioma residua (> 90%) were the most consistent findings, while progressive arteriopathy, headaches, learning differences, and airway involvement emerged as the principal sources of long-term morbidity. The reported case illustrates an unusually severe cerebrovascular phenotype, including neonatal hemorrhagic hydrocephalus, dural venous sinus thrombosis, and a late dural arteriovenous fistula, culminating in ataxic cerebral palsy and mild intellectual disability.

**Conclusions:**

PHACE syndrome requires a multidisciplinary, lifelong follow-up strategy. The presented case underscores that cerebrovascular complications may evolve over years to decades after the initial diagnosis, reinforcing the need for long-term neuroradiological surveillance even after apparent clinical stability.

## Introduction

PHACE syndrome is a sporadic, predominantly female-predominant neurocutaneous disorder first described in 1996 as the association of *P*osterior fossa brain malformations, segmental facial *H*emangiomas, *A*rterial cerebrovascular anomalies, *C*ardiac defects and aortic coarctation, and *E*ye abnormalities (Online Mendelian Inheritance in Man 606519) [1]. Sternal clefting, supraumbilical raphe, and other ventral midline developmental defects are also recognized components, leading some authors to use the extended acronym PHACES [2]. Consensus diagnostic criteria were proposed in 2009 and updated in 2016 with care recommendations that stratify patients by major and minor criteria across each organ system [2]. According to Orphanet estimates cited in recent epidemiological work, the prevalence of PHACE syndrome is below 1 case per million, making it an exceptionally rare condition for which population-level data have historically been limited to small, often single-center, case series [3].

The hallmark cutaneous feature of PHACE syndrome is a large (typically >5 cm), segmental infantile hemangioma (IH) of the face, scalp, or neck, most often following the embryonic facial segments described by Waner and colleagues (frontotemporal, maxillary, mandibular, and frontonasal). While classic, rapidly proliferating IH is the most common presentation, a subset of patients present with infantile hemangiomas with minimal or arrested growth (IH-MAG), a GLUT-1–positive variant that is largely formed at birth and shows little subsequent proliferation [7]. Recognition of segmental facial IH-MAG is clinically important because it can be mistaken for a capillary malformation, with the consequence that an underlying PHACE syndrome may go undiagnosed [6, 7].

Beyond the skin, the extracutaneous manifestations of PHACE syndrome are the principal determinants of morbidity and mortality. Cerebrovascular arteriopathy — including arterial hypoplasia, agenesis, persistent fetal vessels (e.g., persistent trigeminal artery), aneurysmal dilatation, and progressive stenoocclusive disease including moyamoya-pattern vasculopathy — is the most frequent extracutaneous diagnostic criterion, reported in over 90% of well-characterized cohorts [2, 4]. A small subset of patients develop frank moyamoya-pattern vasculopathy that may benefit from surgical revascularization [8]. Structural brain anomalies, most often involving the posterior fossa (Dandy-Walker malformation, cerebellar hypoplasia) and midline structures, are present in roughly half of patients and are strong predictors of long-term neurodevelopmental difficulties [2, 4]. Cardiac and aortic arch anomalies (coarctation of the aorta, aberrant subclavian artery origin, ventricular septal defects) occur in approximately half of patients, ocular anomalies (microphthalmia, coloboma, persistent fetal vasculature, optic nerve hypoplasia) in roughly a quarter to a half, and airway hemangiomas — with potential for life-threatening obstruction — in 40–52% depending on the screening method employed [2, 4, 5].

The widespread adoption of oral propranolol since 2008 has fundamentally changed the management of infantile hemangiomas in PHACE syndrome, dramatically reducing the need for surgical airway intervention, systemic corticosteroids, and laser ablation [5]. Early concerns that non-selective beta-blockade might reduce cerebral perfusion pressure and precipitate stroke in patients with pre-existing cerebrovascular arteriopathy have been largely, though not entirely, allayed by subsequent multicenter safety data [10]. However, propranolol does not address the underlying cerebrovascular, structural, and cardiac anomalies, which require dedicated neuroradiological surveillance, often for life. Long-term outcome data — including quality of life into adulthood — have only recently begun to emerge [4].

The aims of this work are twofold. First, we conducted a systematic review of the literature, following PRISMA guidelines, to synthesize current evidence on the epidemiology, clinical spectrum, airway involvement, cutaneous phenotypes, and long-term outcomes of PHACE syndrome. Second, we report the case of a girl with a particularly severe and evolving cerebrovascular phenotype of PHACE syndrome, illustrating the importance of sustained, multidisciplinary, long-term follow-up even after years of apparent clinical stability.

## Materials and methods

### Protocol and reporting standards

This systematic literature review was conducted and reported in accordance with the Preferred Reporting Items for Systematic Reviews and Meta-Analyses (PRISMA) 2020 statement [12].

### Search strategy

A systematic search was performed across four electronic databases: PubMed, Web of Science, EMBASE, and PsycINFO. Search terms combined controlled vocabulary and free-text keywords related to “PHACE syndrome,” “PHACES,” “infantile hemangioma,” “segmental hemangioma,” “cerebrovascular arteriopathy,” “airway hemangioma,” and “infantile hemangioma with minimal or arrested growth,” combined using Boolean operators. No language or publication-date restrictions were applied at the search stage. The complete search across all databases identified 319 records.

### Eligibility criteria

Studies were eligible for inclusion if they: (1) enrolled human participants with a diagnosis of PHACE or PHACES syndrome based on consensus diagnostic criteria; (2) reported original clinical, epidemiological, or histopathological data (case series, cohort studies, or multicenter retrospective/prospective studies); and (3) reported at least one outcome of interest relevant to diagnosis, clinical phenotype, management, or long-term prognosis. Studies were excluded if they: had a wrong study design (e.g., narrative commentaries without original data, n = 8); enrolled a population not meeting PHACE/PHACES diagnostic criteria (n = 7); did not address an exposure of interest (n = 5); or reported no outcome of interest (n = 11). The remaining full-text exclusions reflected duplication of cohorts, non-extractable data, or overlap with already-included larger series.

### Study selection and data extraction

**Fig. 1:**
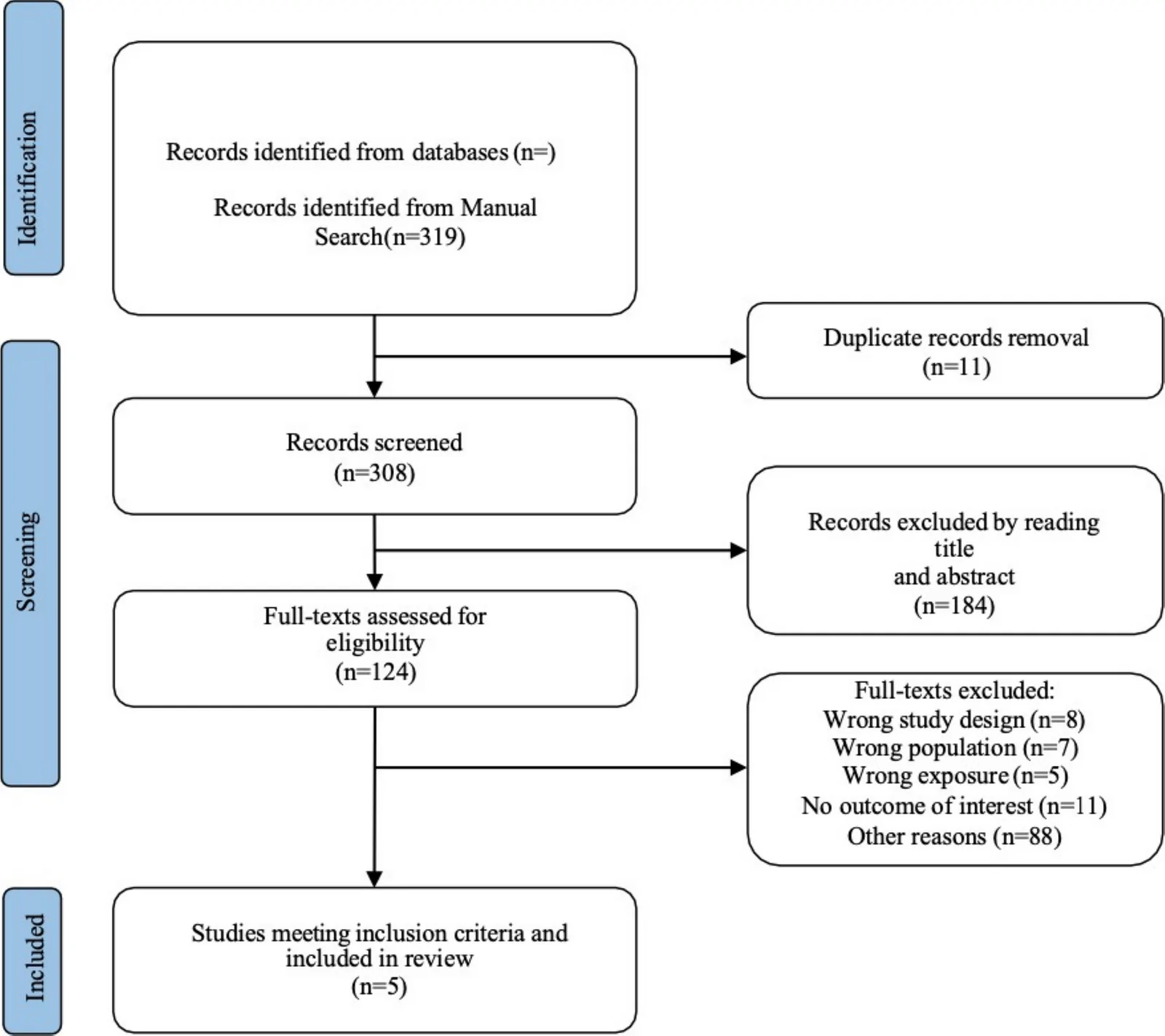
PRISMA 2020 flow diagram of the study selection process

After removal of 11 duplicate records, 308 records were screened by title and abstract, of which 184 were excluded as clearly irrelevant. The remaining 124 full-text articles were assessed for eligibility, and five studies met all inclusion criteria and were retained for qualitative synthesis. The study selection process is summarized in the PRISMA flow diagram (Fig. 1).

### Quality assessment

Given the predominance of case series and retrospective multicenter studies among the eligible literature — an expected finding for a condition with a population prevalence below 1 per million — risk of bias was assessed qualitatively, focusing on case ascertainment methods, completeness of follow-up, and potential for selection or recall bias. All five included studies were judged to provide an acceptable, if inherently limited, level of evidence for a rare disease, with the principal limitations being small sample sizes, retrospective design, and (in two studies) reliance on participant or caregiver self-report.

### Results of the literature review

The five included studies, summarized in Tables 1 and 2, collectively address four complementary aspects of PHACE syndrome: population-based epidemiology and early supportive care needs [3]; long-term, lifespan-spanning health outcomes and quality of life [4]; the prevalence, risk factors, and modern management of airway hemangiomas [5]; and the clinical recognition of infantile hemangiomas with minimal or arrested growth (IH-MAG) as a cutaneous marker of PHACE syndrome [6, 7].

**Table 1:** Characteristics of the five studies included in the systematic review

Author, Year (Ref.)	Country	Study Design	Sample Size (n)	Population/Setting	Primary Aim
Disse et al., 2020 [3]	Germany, Switzerland, Austria	Multinational population- based observational study	19 identified; 10 fully assessed	Children with PHACE syndrome identified via a child- neurologist network (ESNEK)	Estimate population- based prevalence and describe clinical features/early supportive care needs of PHACE in three Europe countries
Braun et al., 2024 [4]	USA, Canada, Spain (multicenter)	Multicenter cross-sectional study with chart review and structured interviews 104 (of 153 contacted)	104 (of 153 contacted)	Individuals ≥ 10 years of age with definite PHACE syndrome from 16 sites	Characterize long-term outcomes and quality of life (PROMIS) of PHACE syndrome in adolescence and adulthood
Czechowic z et al., 2021 [5]	USA (San Francisco, Seattle)	Multicenter retrospective case series	55	Patients with PHACE syndrome evaluated at two multidisciplinar y vascular anomalies clinics (2005–2018)	Describe prevalence, risk factors, and management outcomes of airway hemangiomas in PHACE syndrome
Valdivielso-Ramos et al., 2018 [6]	Spain (5 hospitals)	Multicenter retrospective case series	11	Children with PHACE syndrome whose cutaneous manifestation was an infantile hemangioma with minimal or arrested growth (IH-MAG)	Describe clinical and extracutaneous features of segmental facial IH- MAG as the cutaneous marker of PHACE syndrome
Ma et al., 2017 [7]	Australia (Melbourne)	Photographic/histopathologic case series		Infants presenting after 2000 with birthmarks consistent with IH-MAG, 5 with skin biopsy	Characterize the clinical, histologic, and immunohistochemic al (GLUT-1) features and natural history of IH-MAG

*Abbreviations*: *ESNEK* European child Neurology network, *IH-MAG* Infantile hemangioma with minimal or arrested growth, *PROMIS* Patient-reported outcomes measurement information system

**Table 2:** Main outcomes and clinical relevance of the five studies included in the systematic review

Author, Year (Ref.)	Main Outcomes/Key Findings	Conclusions/Clinical Relevance
Disse et al., 2020 [3]	Prevalence estimates: 6.5/million (Switzerland), 0.59/million (Germany), 0.65/million (Austria) Median age 2.5 years; 9 females, 1 male in detailed cohort Cerebrovascular involvement in 80% of assessed patients Facial hemangioma extent significantly correlated with number of organs involved (p = 0.011) 9/10 patients responded successfully to oral propranolol	First population-based European prevalence data for PHACE syndrome PHACE is likely underreported in Germany and Austria Larger facial hemangioma size may predict greater systemic (multi-organ) involvement, warranting further study
Braun et al., 2024 [4]	IH residua present in 94.1% at median follow-up of 14 years; 89.5% satisfied with cosmetic outcome Cerebrovascular arteriopathy in 91.3%; progression in 29.4% of those with follow-up imaging, with moyamoya/isolated circulation in 8.8% Acute ischemic stroke was rare (1.9%) Headaches/migraines in 72.1%; learning differences in 45.1%; IEP need in 39.4% PROMIS global health scores lower than population norms by ≥ 1 SD; ~ 1/3 reported unaddressed psychological distress	PHACE syndrome carries substantial long-term morbidity extending into adulthood Lifelong multidisciplinary follow-up (neurology, dermatology, mental health, ophthalmology, endocrinology, dental) is essential Surgical resection/ulceration in infancy predicts greater psychosocial impact in adulthood PF/structural brain anomalies are strong predictors of progressive arteriopathy and learning difficulties
Czechowicz et al., 2021 [5]	40% of PHACE patients had documented airway hemangiomas (likely an underestimate) Airway involvement strongly associated with female sex (OR 23), Caucasian ethnicity (OR 5.3), bilateral S3 disease (OR 15–33), and stridor (OR 68) Subglottis (42%) and pharynx (24%) were the most common airway sites Since 2008 (propranolol era), no patient required tracheostomy, laser surgery, or intubation 32% of airway hemangioma patients had no stridor at presentation	Bilateral S3 (“beard”) facial hemangiomas should prompt systematic airway evaluation Propranolol has transformed management of airway hemangiomas from surgical to medical Flexible fiber-optic laryngoscopy is recommended in all PHACE patients, regardless of stridor, given the high rate of asymptomatic airway disease
Valdivielso-Ramos et al., 2018 [6]	Frontotemporal and maxillary segments most frequently involved by IH-MAG Arterial anomalies in 72% (cerebral in 6/11, extracerebral in 5/11); 1 patient had contralateral vascular anomaly Structural brain anomalies in 54%; ocular anomalies in 45%; cardiac anomalies in 3/11 (2 with ventricular septal defect) Ulceration (upper eyelid/upper lip) in 27%, an important diagnostic Clue 4/11 patients had spontaneous resolution; remainder responded to propranolol, corticosteroids, or both Unique associations: fructose intolerance (ALDOB mutation) and retinoblastoma	IH-MAG is a clinically important, often under-recognized cutaneous marker of PHACE syndrome The extracutaneous anomaly profile of IH-MAG-associated PHACE mirrors that of classic IH-associated PHACE Same diagnostic workup (MRI/MRA, echocardiography, ophthalmologic evaluation) and care recommendations apply regardless of hemangioma growth pattern
Ma et al., 2017 [7]	All lesions present at or shortly after birth, with minimal subsequent growth/elevation Characteristic features: telangiectasia, venules, matte erythema with light/dark areas, peripheral pallor or bright papules 5/5 biopsied lesions were GLUT-1 positive, confirming true IH (not capillary malformation) Spontaneous resolution over 12 months to 5 years in most cases without disfigurement Histology showed flat, thin-walled superficial dermal vessels, distinct from proliferative-phase IH	IH-MAG is a distinct, GLUT-1–positive subtype of infantile hemangioma requiring observation rather than early laser treatment Recognition of facial segmental IH-MAG should trigger evaluation for PHACE syndrome Biopsy with GLUT-1 staining is useful when the diagnosis is uncertain, particularly to distinguish IH-MAG from capillary malformations (relevant to Sturge–Weber differential)

*Abbreviations*: *dAVF* Dural arteriovenous fistula, *IEP* Individualized education plan, *IH* Infantile hemangioma, *IH-MAG* Infantile hemangioma with minimal or arrested growth, *OR* Odds ratio, *PF* Posterior fossa, *PROMIS* Patient-reported outcomes measurement information system, *SD* Standard deviation

### Epidemiology and early clinical features

Disse and colleagues conducted the first population-based assessment of PHACE syndrome in Europe, leveraging an established network of child neurologists across Germany, Switzerland, and Austria (the ESNEK network)^3^. Nineteen patients were identified overall, yielding estimated prevalence rates of 6.5 per million in Switzerland, 0.59 per million in Germany, and 0.65 per million in Austria [3]. The markedly higher Swiss estimate, combined with the comparatively low German and Austrian figures, led the authors to conclude that PHACE syndrome is likely underreported in Germany and Austria rather than genuinely less prevalent [3]. In the subset of ten patients with detailed clinical assessment (median age 2.5 years; 9 females, 1 male), cerebrovascular involvement was present in 80%, and the extent of the facial hemangioma correlated significantly with the number of organ systems involved (p = 0.011), a finding that, if confirmed in larger cohorts, could help triage the intensity of extracutaneous screening based on cutaneous severity at presentation [3]. Notably, oral propranolol was successful in treating the facial hemangioma in 9 of 10 patients, and the baseline demographic and cerebrovascular/cardiovascular anomaly rates were consistent with those reported by the US-based PHACE Syndrome International Clinical Registry and Genetic Repository [3].

### Long-term outcomes and quality of life

Braun and colleagues reported the largest and longest-follow-up cohort to date, enrolling 104 of 153 contacted individuals (68%) with definite PHACE syndrome aged ≥10 years (median 14 years, range 10–77 years) across 16 sites [4]. This study combined chart review with structured cross-sectional interviews and, for the first time in this population, applied PROMIS (Patient-Reported Outcomes Measurement Information System) scales to quantify quality of life across pediatric, parent-proxy, and adult domains [4].

Infantile hemangioma residua were present in 94.1% of participants at follow-up, most commonly as color change (71.2%) or textural change (39.4%), with about half having received treatment — predominantly pulsed-dye laser — for these residua [4]. Reassuringly, the large majority reported being “Very Satisfied” or “Satisfied” with their cosmetic outcome (89.5% combined), although a history of surgical resection or infantile ulceration was independently associated with greater self-reported psychosocial impact, even after multivariable adjustment (surgical resection: OR 0.037, 95% CI 0.002–0.813) [4].

Cerebrovascular arteriopathy, present in 91.3% of the cohort, emerged as the most clinically consequential extracutaneous finding over the long term [4]. Among the 68 participants with serial imaging, progressive arteriopathy was identified in 29.4%, at a median age of 10.5 years (range 0.8–19 years), and a subgroup of 8.8% developed moyamoya vasculopathy or progressive stenoocclusion sufficient to produce an “isolated” circulation at or above the level of the circle of Willis [4]. The presence of posterior fossa or other structural brain anomalies was the strongest independent predictor of progressive arteriopathy across multiple multivariable models (odds ratios ranging from approximately 7 to 16 depending on the adjustment set) [4]. Despite this burden of arteriopathy, acute ischemic stroke remained rare, occurring in only 1.9% of the cohort [4].

Neurologic and neurodevelopmental morbidity was substantial: 72.1% reported persistent, troublesome headaches (45.2% with a formal migraine diagnosis), 45.1% reported learning differences, and 39.4% had used or required an individualized education plan [4]. Larger hemangioma size (10–15 cm versus 5–10 cm) was associated with a higher likelihood of headaches (OR 5.5) [4], while posterior fossa/structural brain anomalies, with or without additional diagnostic features, and ocular anomalies were each independently associated with learning differences and the need for educational support [4]. Importantly, arterial anomalies alone — the most common PHACE diagnostic criterion — were *not* significantly associated with learning difficulties, suggesting that structural rather than purely vascular brain involvement drives most of the cognitive burden [4].

Finally, on PROMIS measures, average global health scores were lower than population norms by at least one standard deviation across pediatric, parent-proxy, and adult respondents, with scores comparable to those reported in children with uncontrolled asthma or systemic lupus erythematosus [4]. Nearly one-third of participants reported psychological distress that had not been medically addressed, including diagnosed depression (20.2%) and anxiety (29.8%) [4], underscoring the importance of proactively incorporating mental health screening into long-term PHACE follow-up.

### Airway hemangiomas

Czechowicz and colleagues provided the largest multicenter case series to date on airway involvement in PHACE syndrome, identifying documented airway hemangiomas in 22 of 55 patients (40%), most strongly predicted by bilateral involvement of the S3 (“beard”) facial segment (86% vs. 15% for unilateral disease) [5]. Since the introduction of propranolol in 2008, no patient in this cohort has required intubation, laser resection, or tracheostomy, in contrast to the pre-2008 era [5]. Because roughly one third of patients with confirmed airway hemangioma lacked stridor at presentation, flexible fiber-optic laryngoscopy is recommended as a routine component of the PHACE workup regardless of symptoms [5].

### Infantile hemangioma with minimal or arrested growth (IH-MAG) as a cutaneous marker of PHACE

Two complementary studies addressed IH-MAG, a clinically distinct, GLUT-1–positive variant of infantile hemangioma that is largely formed at birth and undergoes minimal further proliferation [6, 7]. Ma and colleagues characterized the clinical, histologic, and immunohistochemical features of nine such lesions, most located on the limbs or trunk, demonstrating consistent features of telangiectasia, venules, and “matte” erythema with alternating light and dark areas, together with spontaneous resolution over 12 months to 5 years in the majority of cases [7]. On histology, IH-MAG showed flat, thin-walled, GLUT-1–positive vessels in the superficial dermis — morphologically closer to capillary malformation than to a proliferating hemangioma — such that GLUT-1 immunostaining was identified as the key discriminator when the clinical diagnosis was uncertain [7].

Valdivielso-Ramos and colleagues then presented the largest reported series of segmental *facial* IH-MAG occurring specifically in the context of PHACE syndrome (n = 11, 10 girls and 1 boy) [6]. The frontotemporal and maxillary segments were most frequently involved, and ulceration of the upper eyelid or upper lip — present in 27% of patients — served as an important clinical clue prompting further evaluation [6]. Critically, the extracutaneous anomaly profile in this IH-MAG cohort closely paralleled that reported for PHACE with classic, proliferative IH: arterial anomalies in 72% (cerebral in the majority), structural brain anomalies (predominantly cerebellar/posterior fossa) in 54%, and ocular anomalies in 45% [6]. Four of the eleven patients underwent spontaneous resolution of their cutaneous lesion, while the remainder were managed with propranolol, corticosteroids, or both [6]. Two unusual associations — a homozygous *ALDOB* mutation causing fructose intolerance, and a concomitant retinoblastoma — were also reported, broadening the recognized phenotypic spectrum of PHACE-associated IH-MAG [6]. Taken together, these two studies establish that the growth pattern of the cutaneous hemangioma — classic proliferative IH versus IH-MAG — does not predict the severity or distribution of extracutaneous PHACE manifestations, and that the same diagnostic and care pathways should be applied regardless of hemangioma morphology [6, 7].

### Summary Tables

Table 1 summarizes the design, population, and primary aims of the five included studies. Table 2 summarizes their main outcomes and clinical relevance.

## Case report

We report the case of a girl, now 12 years of age, with PHACE syndrome characterized by a left V1-distribution facial hemangioma, multiple ocular and cerebrovascular anomalies, and a complex, evolving neurosurgical and neurovascular course spanning more than a decade.

### Presentation and neonatal history

The patient was born at term via uncomplicated vaginal delivery, following a pregnancy complicated by hyperemesis gravidarum. Family history was notable for cleft palate, epilepsy, and unspecified ocular and hematologic disorders in relatives; the parents were non-consanguineous and healthy. Routine prenatal ultrasound and infectious disease screening were unremarkable, and fetal movements were reported as normal. Birth parameters were within normal limits (weight 3350 g, length 51 cm, head circumference 32 cm), with good Apgar scores and good adaptation to extrauterine life.

**Fig. 2:**
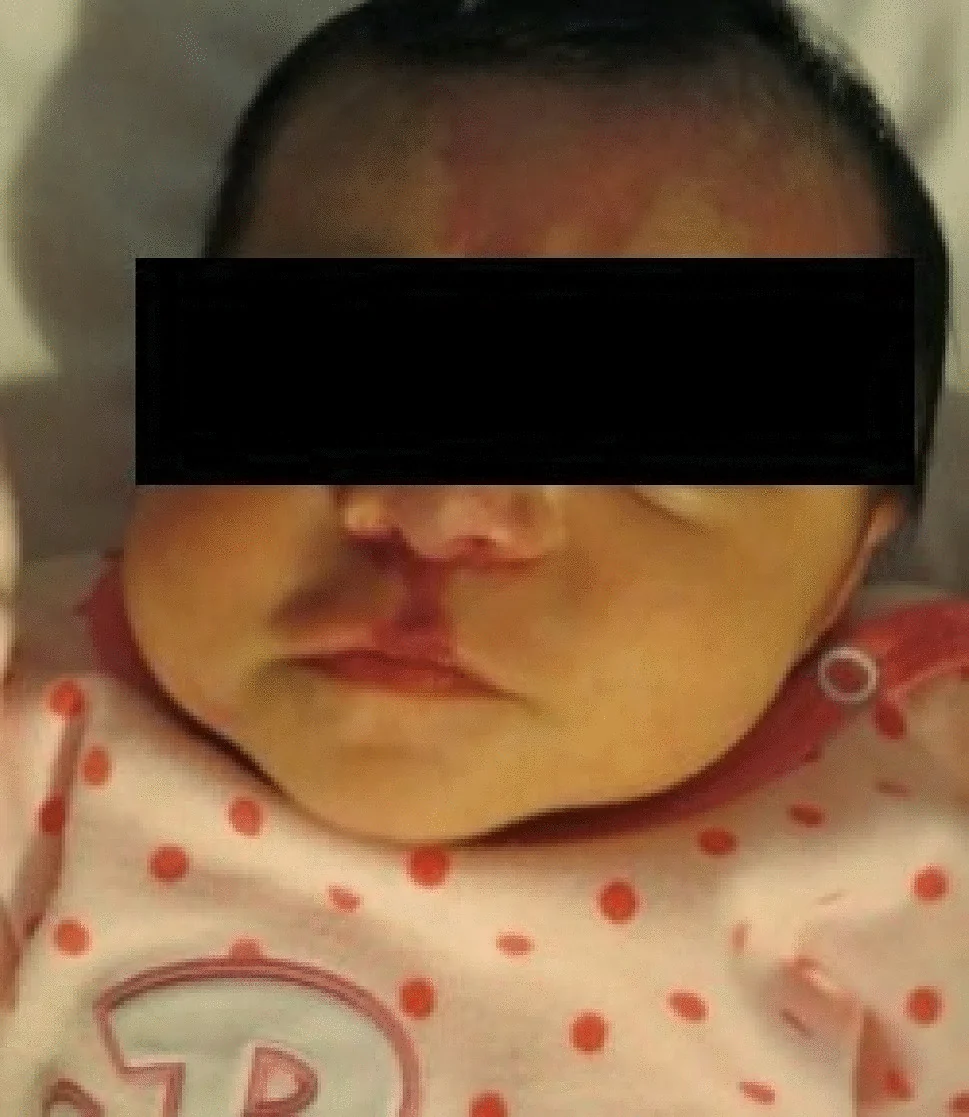
Neonatal facial phenotype showing an extensive segmental hemangioma in the left V1 (ophthalmic) trigeminal distribution, extending to the nasal bridge

Neonatal examination revealed leukocoria of the left eye and an extensive facial hemangioma involving the left first (ophthalmic) branch of the trigeminal nerve, extending to the nasal bridge, together with a small capillary hemangioma of the left thigh and a nevus of the right leg (Fig. 2). Ophthalmologic evaluation demonstrated corneal stromal thinning with neovascularization and a ring-shaped scar.

Brain magnetic resonance angiography (MRA) revealed hypoplasia/aplasia of the A1 segment of the left anterior cerebral artery, a thread-like (hypoplastic) right V4 segment of the vertebral artery with left vertebral dominance, and a vermian developmental venous anomaly draining into the vein of Galen, with generalized hypertrophy of the deep venous drainage system, including hypertrophic superficial venous structures in the right temporo-polar and contralateral temporo-opercular regions. Echocardiography showed a mild aneurysm of the fossa ovale with a small left-to-right shunt, which subsequently resolved on follow-up imaging.

### Neonatal hemorrhage, hydrocephalus, and neurosurgical interventions

At one month of age, the patient was admitted for acute-onset hypotonia and hyporeactivity. Head computed tomography (CT) revealed intraventricular hemorrhage involving the fourth, third, and lateral ventricles with extension into the cerebral aqueduct, marked dilatation of the fourth ventricle, a right paravermian intraparenchymal hematoma with surrounding edema, and subarachnoid hemorrhage of the posterior fossa and basal cisterns, with dilated temporal and occipital horns and effaced cortical sulci consistent with acute hydrocephalus.

Emergent external ventricular drainage (EVD) was placed via the anterior fontanelle. Postoperatively, the patient developed focal clonic seizures of the upper limbs with eye deviation (retroversion of the globes) and sucking movements; these were successfully controlled with intravenous midazolam and fentanyl, with complete resolution of seizure activity.

Subsequent neurological examination revealed absent bilateral corneal and cough reflexes, asymmetric muscle tone (increased on the left), and a posture characterized by trunk convexity to the right with the head rotated rightward. Follow-up CT and MRI confirmed dural venous sinus thrombosis involving the superior sagittal sinus and the right transverse/sigmoid sinus, with ectasia and congestion of the venous structures; this was treated with low-molecular-weight heparin (LMWH).

Because of persistent hydrocephalus — with progressive ventricular enlargement and increasing head circumference — the patient underwent endoscopic third ventriculostomy (ETV). Three months later, a ventriculoperitoneal shunt (VPS) was placed, after which neuroradiological findings remained stable.

The combination of an extensive facial segmental hemangioma in the V1 trigeminal distribution, ocular abnormalities (corneal thinning, neovascularization, leukocoria), multiple cerebrovascular malformations affecting both the venous system (vermian developmental venous anomaly, generalized deep venous hypertrophy, bilateral temporal venous hypertrophy) and the arterial system (hypoplasia of the left A1 anterior cerebral artery segment, thread-like right vertebral artery V4 segment with left dominance), and delayed acquisition of psychomotor milestones raised strong clinical suspicion of PHACE syndrome.

### Long-term follow-up: shunt revision, dural arteriovenous fistula, and recurrent embolization

Following approximately seven years of clinical stability, the patient presented with acute shunt malfunction, manifesting as worsening headache, projectile vomiting, and lethargy. Head CT confirmed ventricular dilatation, and the VPS was revised with placement of an adjustable-pressure valve. Post-revision brain MRI/MRA demonstrated reduced ventricular size but also revealed an irregular ventricular profile (particularly at the level of the temporal horns), “anomalous” hippocampal configuration, thinning of the corpus callosum, diffuse reduction of white matter volume, enlargement of a left bulbo-pontine cyst, and persistent perimesencephalic venous hypertrophy, together with a new ipsilateral anterior frontal developmental venous anomaly.

On subsequent neuroradiological follow-up, a progressive increase in the caliber of both superficial (dural sinus) and deep (medullary vein) venous structures was noted, more pronounced on the left and accentuated after contrast administration. This finding prompted diagnostic cerebral digital subtraction angiography (DSA), which identified a dural arteriovenous fistula (dAVF) involving the anterior third of the superior sagittal sinus, with marked venous congestion of both cerebral hemispheres; venous drainage through the superior longitudinal sinus appeared inefficient owing to congestion from bilateral frontal pial veins.

The right frontal dural arteriovenous fistula was successfully treated by endovascular embolization. Two days after the procedure, the patient developed acute-onset frontal headache and projectile vomiting; emergency CT demonstrated acute hydrocephalus secondary to VPS obstruction, and a new ventriculoperitoneal valve was placed urgently.

**Fig. 3:**
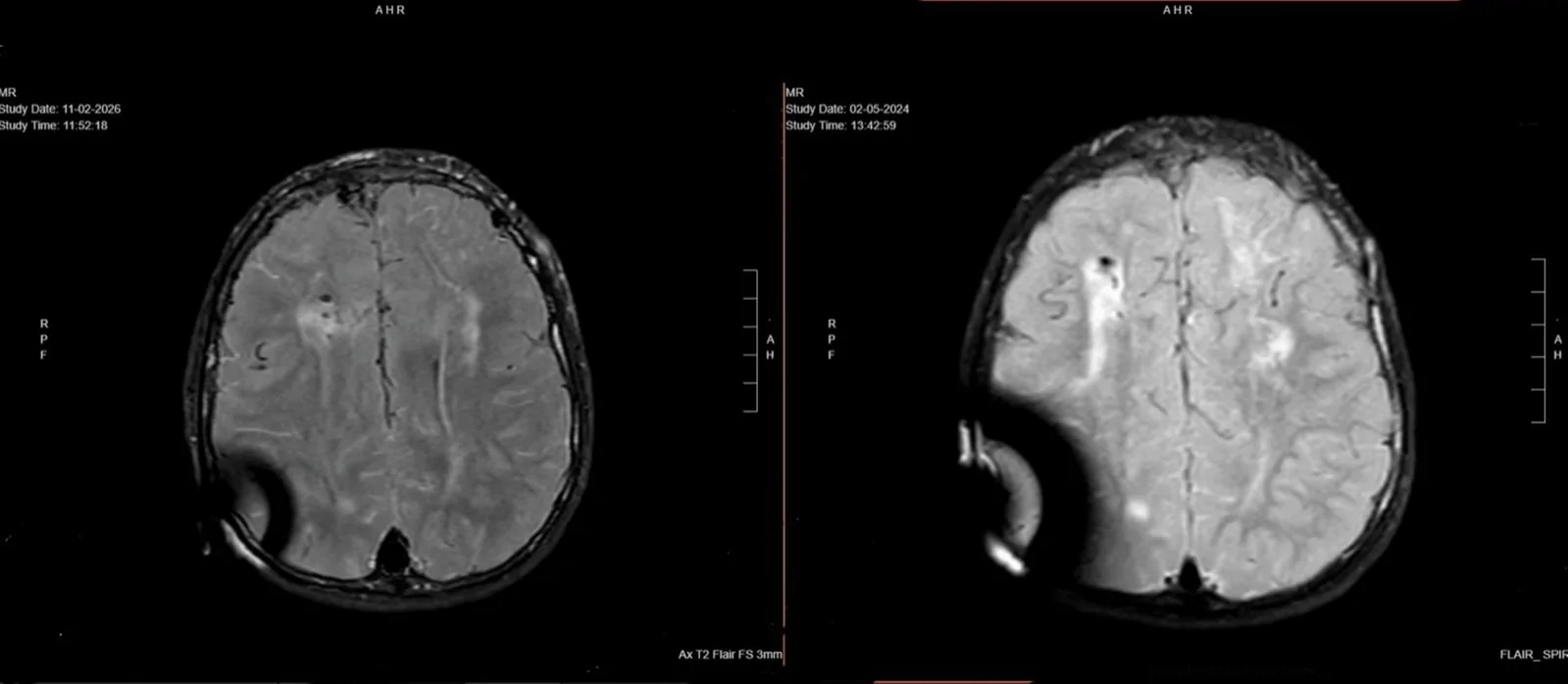
Axial T2-FLAIR MRI at the level of the lateral ventricles, comparing May 2024 (right) and February 2026 (left) studies, showing interval changes in the dural venous sinuses and pial venous structures of both frontal convexities, in keeping with recurrence of the treated vascular malformation

**Fig. 4.**
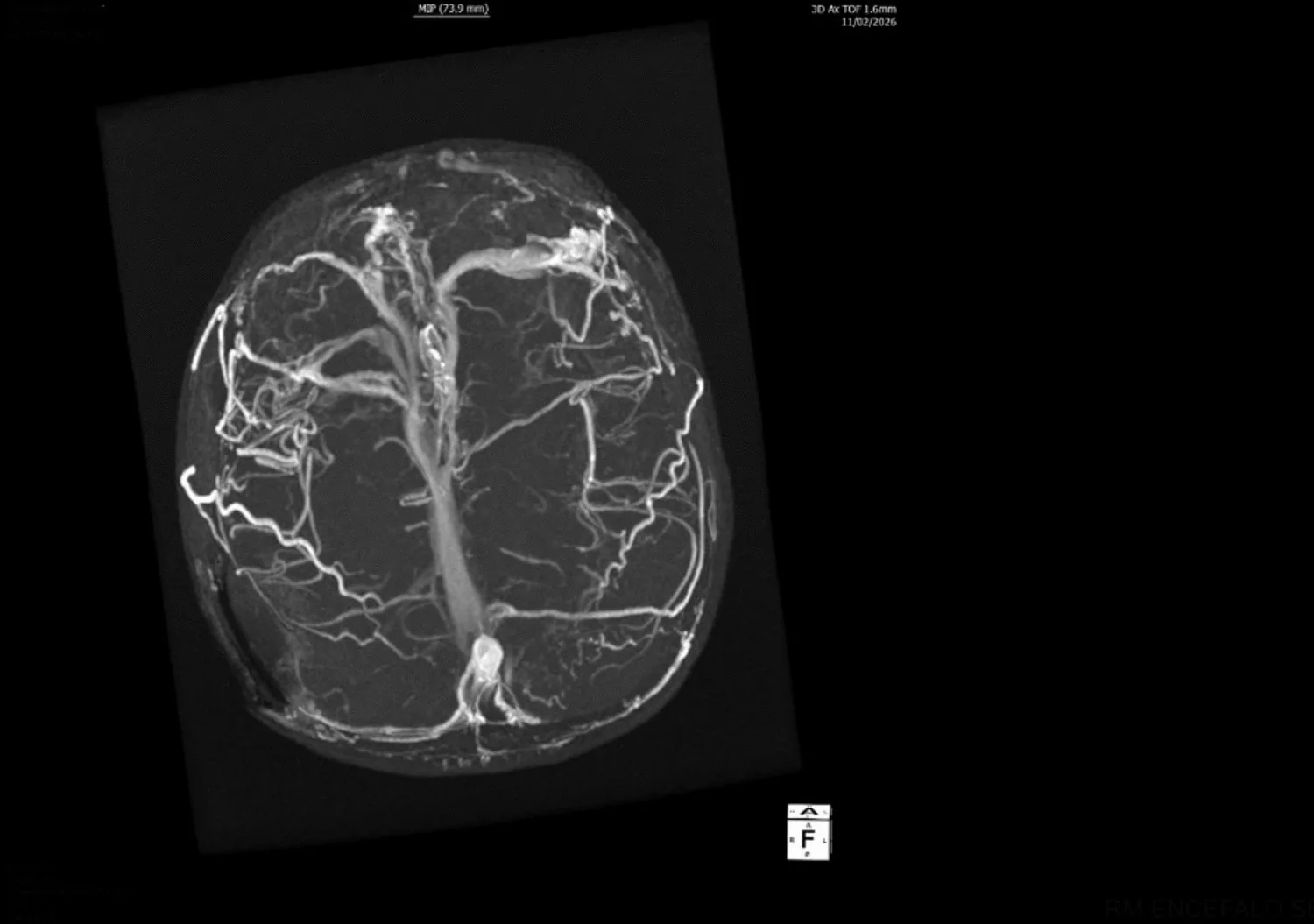
3D time-of-flight MR angiography (February 2026) of the intracranial arterial circulation, obtained as part of the follow-up evaluation

On follow-up DSA, a minor recurrence of the dAVF was identified, supplied by frontal branches of the bilateral middle meningeal arteries with only minimal residual pial venous congestion of the left frontal lobe; this recurrence was managed with a second, uncomplicated embolization of the superior sagittal sinus dural arteriovenous fistula. A subsequent neuroradiological follow-up has detected a further recurrence of the vascular malformation, for which a new diagnostic cerebral angiography is currently pending (Figs. 3 and 4).

### Genetic evaluation

Array comparative genomic hybridization (array-CGH), performed at four months of age, identified a paternally inherited 14q24.1 deletion, classified as non-pathogenic and not considered causally related to the patient’s clinical phenotype. Subsequent clinical exome sequencing identified two variants of uncertain significance (VUS) in simple heterozygosity: one in *RASA1*, a gene associated with capillary malformation–arteriovenous malformation (CM-AVM) syndrome, and one in *SLC2A10*, associated with arterial tortuosity syndrome when biallelic. Neither variant has been definitively linked to the patient’s phenotype, but both are noted given their mechanistic plausibility in a patient with combined capillary malformation and an evolving high-flow dural arteriovenous shunt.

### Vascular anomalies re-evaluation and skin-directed treatment

**Fig. 5:**
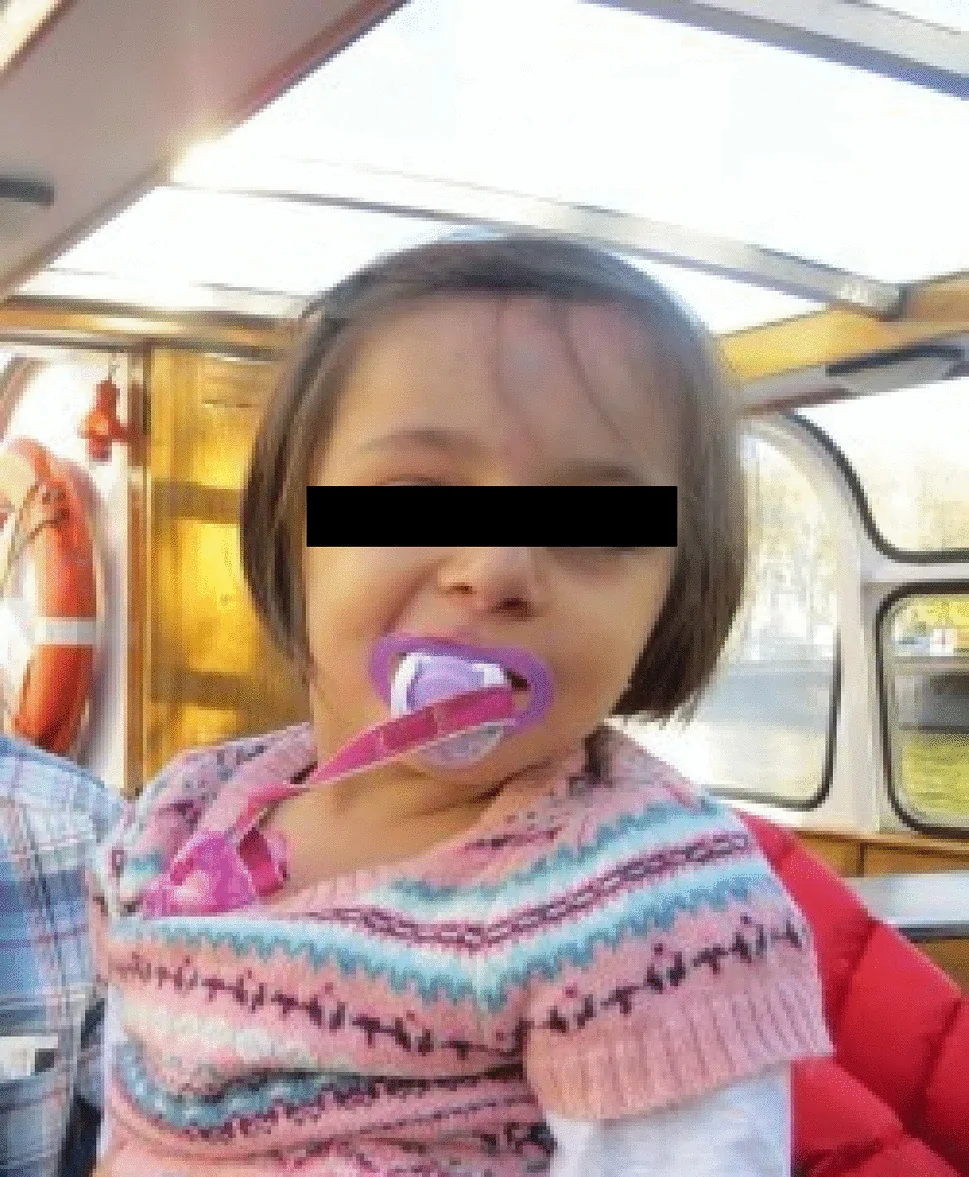
Clinical appearance of the patient at approximately two–three years of age, showing the residual capillary malformation in the left V1 distribution Laser therapy was initiated for the facial capillary malformation. Given the combination of a facial capillary malformation and intracranial vascular anomalies, ongoing neuroimaging surveillance was recommended specifically to exclude Sturge-Weber syndrome and Macrocephaly–Capillary Malformation (M-CM) syndrome as alternative or overlapping diagnoses. The patient also underwent corrective surgery for strabismus of the left eye

At approximately two years of age, the patient was evaluated at a dedicated vascular anomalies and vascular surgery center, which confirmed a midline-extending capillary malformation of the left first trigeminal branch (V1) involving the ipsilateral scalp, together with flat angiomas of the left submandibular region and right thigh (Fig. 5).

### Current functional and neurodevelopmental status

The patient’s current clinical picture is characterized by ataxic cerebral palsy and mild intellectual disability, with a non-verbal IQ of 64, influenced by coexisting visual impairment. Speech is sufficiently intelligible but shows dysarthric features. She demonstrates a good bimanual motor repertoire, although movements are slow and hand-eye coordination is impaired. A bilateral valgus-pronation deformity of the feet is present, for which she wears supramalleolar orthoses (SMOs).

From a functional mobility standpoint, the patient is able to sit independently without support and can perform postural transitions — from the floor or from a wheelchair to standing — independently. However, standing can be maintained only for short periods because of marked postural instability of ataxic origin. Short-distance mobility is achieved via cruising (holding onto furniture or supports), while longer-distance ambulation requires a posterior walker; her gait is characteristically ataxic. The patient continues to receive multidisciplinary neurorehabilitation, including physiotherapy targeting balance, gait, and upper-limb coordination.

### Rehabilitation and functional management

The patient was enrolled in a multidisciplinary neurorehabilitation program (physiotherapy and speech therapy) at 7 months of age, achieving sequential gross motor milestones — independent sitting at 18 months, standing with support and crawling at 2 years, and bottom-shuffling at 3 years — with assisted ambulation progressing from an anterior walker at 4 years to a posterior walker at 6 years, and an established, consolidated gait pattern confirmed on serial gait analysis at age 11.

Following family consultation, outpatient rehabilitation was subsequently redirected toward upper-limb function, using a task- and goal-oriented approach targeting manual dexterity and precision. After a 3-month intensive intervention (25 one-hour sessions), the patient showed significant, sustained improvements in range of motion, dexterity, and fluency on standardized upper-limb assessment (Melbourne Assessment 2), maintained after a subsequent 3-month washout period. Functional gains were corroborated by Goal Attainment Scaling, with the patient progressing from independently unwrapping a sweet to opening packets of crisps and, unexpectedly, tissues, and by consistent reports from parents and teachers of improved manual skills in daily activities at home and school, fully retained at follow-up.

## Discussion

**Fig. 6:**
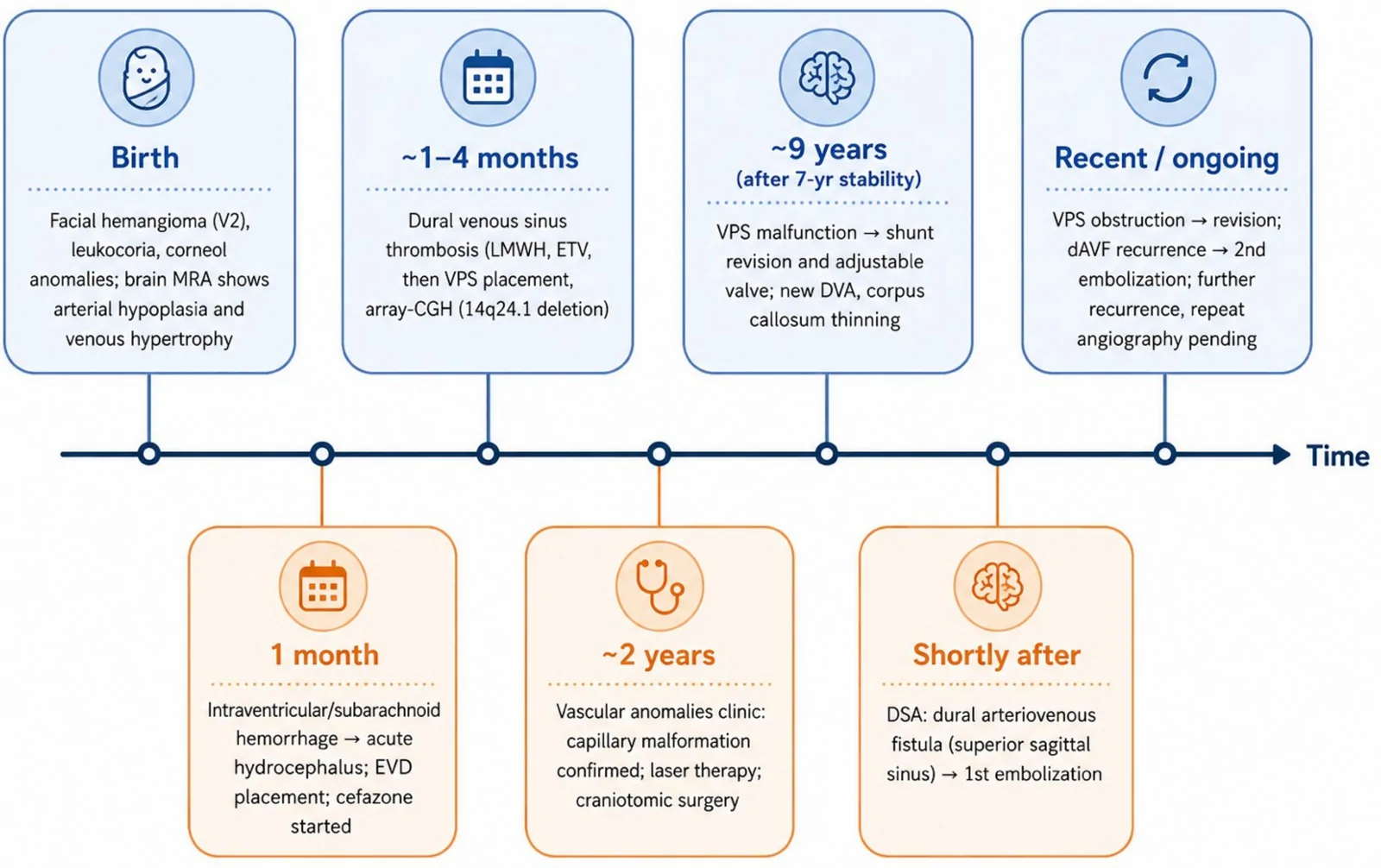
Timeline of major neurovascular and neurosurgical events in the reported case, from birth to the present

This case illustrates several features that are highly representative of the literature reviewed above, while also highlighting an unusually severe and protracted cerebrovascular course (summarized in Fig. 6).

First, the cutaneous presentation — a large segmental hemangioma in the V1 trigeminal distribution with associated leukocoria and corneal abnormalities — is a textbook trigger for PHACE evaluation, consistent with the observation across the reviewed literature that frontotemporal/V1 segmental involvement is strongly associated with both cerebrovascular and ocular anomalies [6]. The presence of a capillary malformation component, later re-characterized at the vascular anomalies clinic, also resonates with the IH-MAG literature reviewed above [6, 7]: although our patient’s lesion was originally identified as a hemangioma, the long-term description of a “capillary malformation” extending to the scalp, combined with the recommendation to exclude Sturge-Weber syndrome, reflects the same diagnostic ambiguity between capillary malformation, IH-MAG, and classic proliferative IH that Ma et al. and Valdivielso-Ramos et al. specifically addressed [6, 7]. As in their cohorts, the precise growth pattern of the cutaneous lesion did not appear to predict the severity of extracutaneous (in this case, cerebrovascular) involvement.

Second, the cerebrovascular phenotype in this patient was unusually severe and multifaceted, encompassing both arterial anomalies (hypoplastic A1 anterior cerebral artery segment, hypoplastic/thread-like vertebral artery segment) and an extensive venous malformation spectrum (vermian developmental venous anomaly, generalized deep venous hypertrophy, bilateral temporal venous hypertrophy), culminating in neonatal intraventricular and subarachnoid hemorrhage with hydrocephalus requiring sequential EVD, ETV, and VPS placement. While Braun et al. reported that arterial cerebrovascular anomalies were present in over 90% of their long-term cohort and that progressive arteriopathy developed in nearly 30% of those with serial imaging [4], neonatal hemorrhagic presentations of the severity seen in our patient are less commonly emphasized in the larger cohort studies, which tend to focus on arterial stenoocclusive progression and moyamoya-pattern vasculopathy rather than venous anomalies and early hemorrhagic hydrocephalus. Notably, a dedicated series of PHACE patients with moyamoya-pattern vasculopathy demonstrated that surgical revascularization (pial synangiosis) can be both radiographically and clinically effective, although such patients — like ours — were exclusively female and often required complex perioperative planning because of overlying facial hemangiomas [8]. This case therefore adds to the relatively sparse literature on venous-predominant cerebrovascular phenotypes in PHACE syndrome.

Third, and perhaps most strikingly, the patient’s clinical course demonstrates that cerebrovascular complications in PHACE syndrome can emerge or evolve *years to decades* after the initial presentation. After seven years of apparent neuroradiological stability, the patient developed a dural arteriovenous fistula of the superior sagittal sinus — a high-flow vascular shunt that required two separate endovascular embolization procedures and has since shown further recurrence. Pediatric dural arteriovenous fistulae are themselves exceedingly rare, and in one of the few dedicated case series, PHACE was specifically identified as one of the underlying vascular-anomaly syndromes associated with this lesion, alongside PTEN-related disorders [9], lending further support to a mechanistic link between PHACE-associated venous anomalies and acquired dural shunting. This evolution underscores a key message echoed by Braun et al.: long-term, lifelong neuroradiological surveillance is essential in PHACE syndrome, even in patients who have been clinically stable for prolonged periods, because the underlying vasculopathy is a *dynamic*, not static, process [4]. The development of a dAVF in the setting of pre-existing venous hypertrophy and developmental venous anomalies also raises the hypothesis — which warrants further study — that congenital venous outflow anomalies in PHACE syndrome may predispose to the later development of acquired or unmasked arteriovenous shunting, possibly related to chronic venous hypertension or remodeling around prior thrombosis (the patient had documented dural venous sinus thrombosis in the neonatal period, treated with low-molecular-weight heparin).

Fourth, the neurodevelopmental outcome in this patient — ataxic cerebral palsy with mild intellectual disability (non-verbal IQ 64) and visual impairment — is consistent with the strong association reported by Braun et al. between posterior fossa/structural brain anomalies and long-term learning differences and developmental delay [4]. In their cohort, four participants (3.8%) had severe developmental delay impacting daily life, and all four had posterior fossa or structural brain anomalies [4]; our patient’s extensive cerebellar/bulbo-pontine involvement (left bulbo-pontine cyst, vermian anomalies) and corpus callosum thinning fit well within this pattern. The ataxic gait pattern specifically reflects the cerebellar and brainstem involvement demonstrated on serial imaging, and the multidisciplinary rehabilitation program described — targeting balance, gait, and bimanual coordination — mirrors the recommendations from Braun et al. for individualized, long-term rehabilitative and educational support in patients with PF/structural brain anomalies [4].

Fifth, the genetic findings in this case — a non-pathogenic paternally inherited 14q24.1 deletion and two variants of uncertain significance in *RASA1* and *SLC2A10* — illustrate the current state of genetic understanding of PHACE syndrome: while a definitive monogenic cause has not been established for the great majority of patients, overlapping or mimicking conditions with known genetic bases remain important differential considerations. Heterozygous *RASA1* mutations cause capillary malformation-arteriovenous malformation (CM-AVM) syndrome, an autosomal dominant disorder characterized by multifocal atypical capillary malformations together with fast-flow arteriovenous malformations or fistulas [11], a phenotypic combination that bears a striking resemblance to our patient’s capillary malformation plus dural arteriovenous fistula. Biallelic *SLC2A10* mutations, by contrast, cause arterial tortuosity syndrome. Although neither variant has been confirmed as causative in this patient, their presence in a child with combined capillary malformation and high-flow vascular anomalies such as the dAVF observed here underscores the importance of considering CM-AVM and related RASopathies in the differential diagnosis and, potentially, in future genetic counseling. The recommendation to exclude Sturge-Weber syndrome and Macrocephaly-Capillary Malformation syndrome on an ongoing basis further reflects the broad differential diagnosis that must be considered and periodically revisited in patients with overlapping capillary, venous, and arterial vascular anomalies of the head and neck.

Finally, this case did not feature clinically apparent airway involvement, despite the V1-predominant facial segment distribution. While Czechowicz et al. found that bilateral S3 (“beard”) involvement was the strongest anatomical predictor of airway hemangioma [5], our patient’s lesion was predominantly frontotemporal/V1 rather than mandibular/S3, which is consistent with the lower airway involvement rates reported for S1-predominant distributions in their series (30% for S1 involvement versus 86% for bilateral S3 involvement) [5]. Nonetheless, given that 32% of airway-involved patients in their cohort lacked stridor [5], ongoing vigilance for subtle airway symptoms (e.g., feeding difficulties, positional stridor, or recurrent croup-like episodes) remains warranted throughout childhood.

## Conclusion

PHACE syndrome is a rare but clinically significant neurocutaneous disorder in which the cutaneous hemangioma — whether classic proliferative IH or IH-MAG — serves primarily as a sentinel marker for a much broader spectrum of cerebrovascular, structural brain, cardiac, ocular, and airway anomalies. The literature synthesized here demonstrates that: (1) PHACE syndrome is likely underdiagnosed in several European populations [3]; (2) long-term morbidity — driven principally by progressive cerebrovascular arteriopathy, headaches, and learning differences — persists well into adulthood and is associated with quality-of-life impairments comparable to those seen in chronic systemic diseases [4]; (3) airway hemangiomas, present in roughly 40% of patients and strongly associated with bilateral S3 facial involvement, warrant systematic screening with flexible laryngoscopy regardless of stridor [5]; (4) IH-MAG is a clinically important, GLUT-1–positive cutaneous variant whose recognition — particularly when segmental and facial — should prompt the same diagnostic workup as classic proliferative IH in suspected PHACE syndrome [6, 7]; and (5) although high-flow vascular complications such as moyamoya-pattern vasculopathy or dural arteriovenous fistulae are rare, both are well documented in PHACE syndrome and can be amenable to surgical or endovascular treatment when promptly recognized [8, 9].

The case reported here adds to this body of evidence by illustrating a severe, venous-predominant cerebrovascular phenotype complicated by neonatal hemorrhagic hydrocephalus and, more than seven years later, by the de novo development of a dural arteriovenous fistula requiring repeated embolization. This trajectory reinforces the central message that PHACE syndrome demands a lifelong, multidisciplinary follow-up strategy — encompassing neurology, neurosurgery, neuroradiology, dermatology, ophthalmology, cardiology, genetics, and rehabilitation medicine — because the underlying vasculopathy may remain clinically silent for years before manifesting as a new, potentially serious complication. Continued long-term, ideally prospective and multicenter, follow-up studies will be essential to refine surveillance protocols, particularly regarding the optimal frequency and modality of neurovascular imaging in patients who have achieved apparent clinical stability. Taken together, these findings support a simple clinical take-home message: in PHACE syndrome, cerebrovascular stability should never be assumed to be permanent, and individualized, lifelong neuroradiological surveillance remains warranted regardless of the interval since diagnosis or the duration of apparent quiescence.

## Data Availability

The datasets generated and analyzed during the current study are not publicly available due to privacy and ethical restrictions concerning patient data, but may be available from the corresponding author upon reasonable request and subject to appropriate ethical and data protection requirements.
